# Supplementary material for: Physical activity level and physical fitness in subjects with chronic musculoskeletal pain: a cross-sectional study
Source: PeerJ. 2024 Feb 6;12:e16880. doi: 10.7717/peerj.16880 (PMC10854395; doi:10.7717/peerj.16880)
Supplement: Supplemental Information 3 [file peerj-12-16880-s003.pdf]

Generalitat de Catalunya
Departament de Sanitat i Seguretat Social
Direcció General de Salut Pública

Encuestador/a ☐☐ **Número de cuestionario** ☐☐☐☐☐

Nombre .... ✍

Apellidos .... ✍

CUESTIONARIO DE ACTIVIDAD FÍSICA

Nos interesa conocer el tipo de actividad física que la población realiza en su vida diaria. Este estudio forma parte de un estudio a gran escala que se lleva a cabo en muchos países del mundo. Sus respuestas nos permitirán comparar la actividad física que se realiza en Cataluña respecto a la gente de otros países.
Las preguntas se refieren al tiempo que ha empleado haciendo actividad física en **los últimos siete días**. Se incluyen preguntas sobre la actividad física que realiza en el trabajo, en casa o en el jardín, para desplazarse de un lugar a otro, y en su tiempo libre, si hace actividad física de ocio, ejercicio físico o practica algún deporte.

Sus respuestas son importantes.

Por favor, conteste todas las preguntas aunque no se considere una persona físicamente activa.

MUCHAS GRACIAS POR PARTICIPAR-

Al contestar las preguntas, recuerde que

- La actividad física **vigorosa** se refiere a aquellas actividades que le suponen un esfuerzo físico duro y le hacen respirar mucho más fuerte de lo normal.

- La actividad física **moderada** se refiere a aquellas actividades que le suponen un esfuerzo físico moderado y que le hacen respirar algo más fuerte de lo normal.

1a. En los últimos 7 días, ¿cuántos días ha realizado actividad física **vigorosa** como levantar pesos pesados, cavar, trabajo intenso en la construcción, clases de aeróbic o similar o bicicleta a ritmo rápido?

Piense *sólamente* en aquella actividad física que duró como mínimo 10 minutos.

☐ **días a la semana** ⇒

o bien

☐ **ningún día a la semana.**

1b. En total, ¿cuanto tiempo suele estar haciendo actividad física vigorosa en uno de estos días?

☐ **horas** ☐ **minutos**

2a. De nuevo, piense *sólo* en aquella actividad física que duró como mínimo 10 minutos. En los últimos 7 días, ¿cuántos días ha realizado actividad física **moderada** como transportar pesos ligeros, bicicleta a ritmo regular, dobles de tenis?. No tenga en cuenta el caminar.

☐ **días a la semana**

o bien

☐ **ningún día a la semana**

2b. En total, ¿cuanto tiempo suele estar haciendo actividad física moderada en uno de estos días?

☐ **horas** ☐ **minutos**

3a. En los últimos 7 días, ¿cuántos días ha **caminado** al menos 10 minutos?. Cuente si camina en su trabajo y en casa, para ir de un sitio a otro y si camina en su tiempo de ocio, como deporte, o para hacer ejercicio o para pasar el tiempo.

☐ **días a la semana**

o bien

☐ **ningún día a la semana**

3b. En total, ¿cuanto tiempo suele estar caminando en uno de estos días?

☐ **horas** ☐ **minutos**

La última pregunta se refiere al tiempo que está **sentado** en los días laborables en el trabajo, en casa, mientras asiste a clase, y en su tiempo libre. Cuente el tiempo que está sentado en el despacho, de visita en casa de amigos, leyendo cuando viaja en autobús o sentado o tumbado viendo la televisión.

4. En los últimos 7 días, ¿cuánto tiempo en total ha estado ***sentado*** en un día **laborable**?

☐ **horas** ☐ **minutos**

Aquí se acaba el cuestionario, muchas gracias por participar
